# Investigation of genetic variation and lifestyle determinants in vitamin D levels in Arab individuals

**DOI:** 10.1186/s12967-018-1396-8

**Published:** 2018-01-30

**Authors:** Massimo Mezzavilla, Sara Tomei, Fadi Alkayal, Motasem Melhem, Maisa M. Ali, Monira Al-Arouj, Abdullah Bennakhi, Osama Alsmadi, Naser Elkum

**Affiliations:** 1 Out-Patient Clinic, 5th Floor, Sidra Medicine, Qatar Foundation, Al Luqta Street, Education City North Campus, PO Box 26999, Doha, Qatar; 2 Dasman Diabetes Institute, Jasim Mohamad Al Bahar St, Kuwait City, Kuwait; 3 King Hussein Cancer Center, Amman, Jordan

**Keywords:** Vitamin D, Arab population, GC gene, Recursive partitioning analysis

## Abstract

**Background:**

Differences in the concentrations of circulating 25-hydroxyvitamin D [25(OH)D] are associated with a wide range of health outcomes; however, most studies on genetic variants that impact 25(OH)D levels have been conducted in European populations. Here we aimed to identify common genetic variants that affect vitamin D concentrations in individuals of self-reported Arab ethnicity.

**Methods:**

The study included 1151 Arab subjects living in Kuwait. Common variants of single-nucleotide polymorphisms and genes previously associated with vitamin D levels, such as GC, PDE3B, CYP2R1, and NADSYN1, were genotyped. Raw vitamin D level data were corrected for age, body mass index, and sex and then normalized. Regression tree analyses were performed to identify the impact of genetic variants on vitamin D levels.

**Results:**

Compared with other gene variants, the GC gene variants exhibited the greatest impact on vitamin D levels in our study population, of which rs2298850 had the lowest p value (0.003). Individuals homozygous for the derived allele C had lower vitamin D levels. Analyses of the interaction between the number of years for which the subjects had lived in Kuwait and genetic variation in the GC gene showed that those with the CC genotype of rs2298850 who had lived in Kuwait for < 51 years had a mean 25(OH)D level of 10 ng/ml, whereas those who were homozygous for the ancestral allele had a mean 25(OH)D level of 17 ng/ml. Furthermore, subjects who had lived in Kuwait for > 51 years had higher vitamin D levels (mean 28 ng/ml) regardless of the genotype of their GC gene.

**Conclusions:**

The GC gene may play a major role in determining vitamin D levels in Arab populations.

**Electronic supplementary material:**

The online version of this article (10.1186/s12967-018-1396-8) contains supplementary material, which is available to authorized users.

## Background

Vitamin D deficiency occurs worldwide and is a common public health problem. Previous studies have shown that vitamin D deficiency is associated with the risk of developing osteoporosis, type 1 diabetes, cardiovascular diseases, asthma, and even certain types of cancer [1–6].

Vitamin D levels are dependent on skin pigmentation. Consequently, African Americans are more susceptible to vitamin D deficiency because their darker skin tone limits the penetration of ultraviolet light, in turn reducing the cutaneous synthesis of vitamin D. Ethnic differences in the prevalence of common genetic polymorphisms are another likely explanation for the low vitamin D levels found in African Americans [7–9]. Socio-cultural factors can also affect vitamin D levels. For example, most women in Arab Gulf countries wear veils and are rarely exposed to sunlight, which reduces vitamin D synthesis in the body [10]. Despite the emergence of new data on the high prevalence of vitamin D deficiency, most studies on the genetic and environmental factors affecting vitamin D status have focused on Western populations, and their conclusions do not necessarily apply to populations in different geographic regions. Given the lack of substantial data on genetic and environmental risk factors in populations of Middle Eastern origin in which the prevalence of vitamin D deficiency is high, we sought to determine the genetic and lifestyle risk factors underlying vitamin D deficiency in Arabs.

Recent genetic studies have associated vitamin D deficiency with several candidate genes, including the cytochrome P450, family 2, R (CYP2R1) gene; the group-specific component (GC) gene, and the 7-dehydrocholesterol reductase/NAD synthetase 1 (DHCR7/NADSYN1) gene. These genes are involved in hydroxylation, vitamin D transport, and cholesterol synthesis, respectively. The best indicator of vitamin D levels in humans is the serum concentration of its main circulating metabolite, 25-hydroxyvitamin D [25(OH)D]. The association between polymorphisms in these genes and 25(OH)D levels has been previously studied in African Americans and found that a CYP2R1 SNP, rs12794714, exhibited the strongest signal of association [11], and also among 30,000 individuals of European descent [12] where variants near genes DHCR7 (CYP2R1, CYP24A1), and GC identified to influence vitamin D status.

This study aimed to examine the effect each of the genes listed above has on vitamin D levels in a group of Arabs living in Kuwait for various lengths of time. Our main objective was to examine the influence of lifestyle factors and genetic variation on vitamin D status.

## Methods

### Study subjects

This cross-sectional study included 1151 Arab adults (age > 18 years) living in Kuwait. These subjects were part of a large multi-ethnic cohort that was randomly selected as previously described [13]. Our subjects were randomly selected from the computerized registry of the Public Authority of Civil Information. The study conformed to the principles outlined in the Declaration of Helsinki and was approved by the Scientific Advisory Board and Ethical Review Committee at Dasman Diabetes Institute. Informed written consent was obtained from all subjects before their enrolment in the study.

### Phenotypic data

Physical and anthropometric measurements, including body weight, height, and waist circumference, of all subjects were recorded. Blood samples were obtained after at least 10 h of overnight fasting and were analyzed for fasting blood glucose, hemoglobin A1c, and fasting insulin levels as well as for lipid profiles that included triglycerides, total cholesterol, and low- and high-density lipoprotein levels. Glucose levels and lipid profiles were measured using a Siemens Dimension RXL chemistry analyzer (Diamond Diagnostics, Holliston, MA, USA). Serum 25(OH)D levels were measured with a chemiluminescent competitive immunoassay using a DiaSorin LIAISON analyzer (DiaSorin Inc., MN, USA) following the manufacturer’s protocol. The intra-assay coefficients of variation were 5.5 and 4.0% at 10 and 25 ng/ml, respectively, and the inter-assay coefficients of variation were 8 and 6% at 15 and 40 ng/ml, respectively.

### DNA isolation and genotyping

From each subject, 4-ml blood was drawn into ethylenediaminetetraacetic acid tubes. DNA was isolated using commercial kits following the manufacturer’s recommendations as previously reported [13]. DNA quantity and quality were checked using an Epoch Microplate Spectrophotometer before genotyping.

Genotyping was performed with TaqMan on an Applied Biosystems 7500 Real-Time PCR System as previously reported [13]. After polymerase chain reaction amplification, an endpoint plate read was performed. Fluorescence measurements obtained during the plate read were used in Sequence Detection System software to plot fluorescence values based on the signals from each single plate well. The results were plotted on a two-dimensional scatter plot of the major versus minor allele. Genotyping calls were assessed based on the allele discrimination plots and manually reviewed by examining the single amplification plots.

### Statistical analyses

The effects of various parameters were investigated using linear regression analyses as implemented in the R environment. Vitamin D levels of all subjects were adjusted for sex, age, number of years in Kuwait, triglyceride levels, total cholesterol levels, and hip circumference. The residuals were normalized using the rntransform function implemented in the GenABEL package [14] that performs quantile normalization of residuals from a generalized linear model analysis.

In addition, conditional inference-based recursive partitioning (implemented in the R “party” package) [15] was used to determine the influence of the genetic variables on normalized vitamin D levels (as a quantitative trait). This approach searched predictor variables having main effects and higher-order interactions. Stepwise modeling and splitting was applied to produce a classification tree that showed how each genotype affected vitamin D levels. This approach was divided into two phases: in the first phase, we analyzed each gene separately, highlighting which single-nucleotide polymorphism (SNP) had the largest effect on vitamin D levels. In the second phase, we constructed a regression tree using only the SNPs identified as significant in the phase 1 analyses after Bonferroni correction. The final tree is based on the splitting variables of each node with the highest statistical significance. Regression trees allows to construct an hierarchy of the variables with the first node corresponding to the variable with the strongest effect, the second and the third nodes correspond to variables with significant impact of the phenotype. Recursive partitioning is widely used in medicine and genetics to describe interaction between variables.

The genotype distributions and derived allele frequencies of the SNPs of interest were compared with similar distributions from the reference dataset of 1000 Genomes Project. The distribution of the combined annotation-dependent depletion (CADD) score for each variant was obtained [16].

## Results

The descriptive characteristics of all subjects are summarized in Table 1. In total, 1151 subjects (702 men and 449 women), with mean age of 46 (5th percentile: 27, 95th percentile: 64) years and a mean of 35 (5th: 5, 95th: 61) years of living in Kuwait, were analyzed. The mean 25(OH)D level in our cohort was 12.24 (5th: 4.2, 95th: 44.5) ng/ml.

**Table 1:** Clinical and biochemical profiles for study subjects stratified by gender

Parameters	Overall (n = 1151)	Male (n = 702)	Female (n = 449)
Age, years	36 (11.6)	45.6 (11.6)	46.3 (11.6)
Height (cm)	166.7 (9.1)	172 (6.6)	158.5 (5.67)
Weight (kg)	87.4 (18)	90.8 (17.6)	82 (17.2)
BMI (kg/m^2^)	31.4 (6)	30.7 (5.5)	32.6 (6.54)
Waist-circumference (cm)	101.2 (13.17)	102.53 (12.9)	99.1 (13.34)
Hip	110.2 (11.23)	108.4 (10.8)	113.18 (12.7)
FBG (mmol/l)	6.24 (2.63)	6.4 (2.8)	5.9 (2.3)
HbA1c (DCCCT%)	6.1 (1.63)	6.3 (1.72)	5.9 (1.4)
Triglycerides (mmol/l)	1.6 (0.94)	1.75 (1.04)	1.37 (0.72)
Total cholesterol (mmol/l)	5.12 (1.03)	5.1 (1)	5.12 (1.01)
LDL cholesterol (mmol/l)	3.3 (0.95)	3.4 (0.97)	3.23 (0.94)
HDL cholesterol (mmol/l)	1.1 (0.32)	1.01 (0.25)	1.29 (0.34)
25(OH)D (ng/l)	17.7 (14.4)	15 (12.3)	22 (16)
Duration in Kuwait (years)	32.9 (17.3)	30 (17.4)	36.6 (16.7)

Mean and standard deviation are reported

**Fig. 1:**
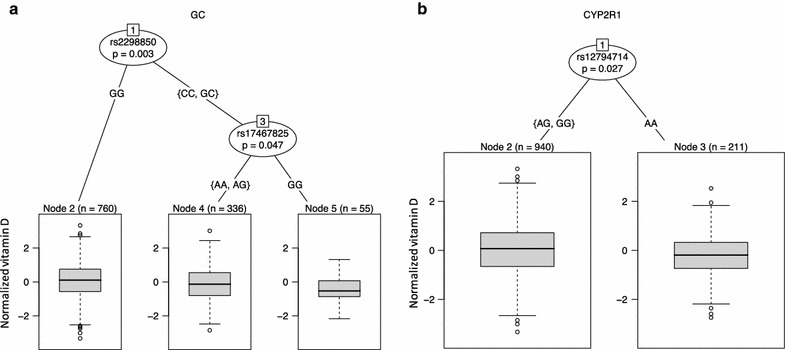
Regression tree for each gene in Arabs. **a** Regression tree analyses for variation in GC gene, **b** regression tree analyses for variation in CYP2R1. The p values are corrected for Bonferroni

Single-gene tree regression analyses showed that only two genes (GC and CYP2R1) were significantly associated with vitamin D levels in our cohort (Fig. 1a, b). The associated SNP rs12794714 in CYP2R1 had a CADD score of > 10, whereas all other variants had low CADD scores of < 7.9 (Additional file 1: Table S1). Partitioning tree analyses revealed that this association occurred in the GC gene. The analyses showed that only the variants in GC gene had a significant effect after Bonferroni correction.

**Fig. 2:**
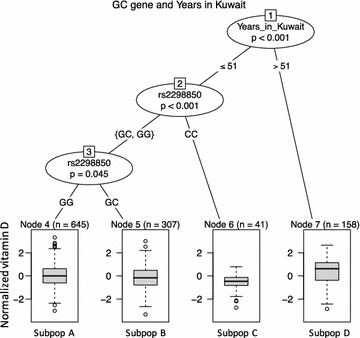
Partitioning tree analyses of GC gene variation and number of years living in Kuwait in Arabs individuals. The p values are corrected for Bonferroni

Multi-regression analysis showed that among lifestyle and environmental factors, the number of years the subjects had lived in Kuwait had a strong impact on their vitamin D levels (p = 3.98e−06). Combining the genetic information with this parameter in a partitioning tree analysis, we discovered that subjects who had lived in Kuwait for > 51 years had vitamin D levels [mean (SD) raw level, 28 ± 15 ng/ml] higher than those in subjects who had lived in Kuwait for < 51 (16 ± 13) years. Vitamin D levels (10 ± 6 ng/ml) were lowest in subjects who had a homozygous CC genotype of rs2298850 compared with subjects who were heterozygous (15 ± 13 ng/ml) or had a homozygous GG genotype (17 ± 13 ng/ml; Fig. 2).

The tree analyses revealed four diverse groups (designated subpopulations A–D) corresponding to subpopulations with various percentages of subjects who could be considered vitamin D deficient according to cut-offs of 12 and 20 ng/ml. The vitamin D level classifications are summarized in Table 2.

**Table 2:** Categorization of individuals according to raw values of vitamin D and regression tree analyses

Group	% Deficient (< 12) ng/ml	% Insufficient (12–20) ng/ml	% Sufficient (> 20) ng/ml
SubpopA	47	25	28
SubpopB	57	23	22
SubpopC	70	25	5
SubpopD	22	11	67

## Discussion

Vitamin D status is a complex trait, and to date, most studies investigating circulating 25(OH)D levels have been conducted on Caucasian and Western populations. A meta-analysis showed that compared with Caucasian, non-Caucasian have lower vitamin D levels [17]. Other studies have shown that compared with Caucasians, Gujarati Indians in West London [18] and African and Mexican Americans [19] have lower plasma levels of vitamin D.

In the present study, regression tree analyses of normalized 25(OH)D levels using SNPs single and multiple genes revealed that the GC gene is the major actor in Arabs. The GC gene, which belongs to the albumin family, helps vitamin D activity by carrying vitamin D metabolites [7]. A major role of the GC gene has also been observed in French populations [20]. In our Arab cohort, the variant with the lowest p value was rs2298850. Notably, this SNP is an intronic variant with a CADD score of < 10, which indicates a low likelihood of deleteriousness. In our dataset, only rs12794714 had a CADD score of > 10 [16], which can be considered a cut-off to discriminate between deleterious and non-deleterious variants. This variant is the most significant for the CYP2R1 gene in Arabs and is also an expression quantitative trail locus for the COPB1 gene, which has been associated with vitamin D levels in children [21]. The effects of the variants in other genes were not significant compared with those of rs2298850 in regression tree analyses.

The results of our study also showed that the number of years subjects had lived in Kuwait had a positive impact on vitamin D levels. We hypothesized that this outcome results from acclimation to sun exposure, i.e., subjects tend to avoid sunlight initially but less so in later years. This lifestyle parameter interacted with genetic background but only in subjects who had lived in Kuwait for < 51 years. In the regression tree analysis, this outcome indicated a dominant effect of the lifestyle parameter over the underlying genetic variation. However, genetic variation in the GC gene represents a risk factor for vitamin D deficiency in Arabs, particularly those who are homozygous for the C allele. While the CC genotype showed significance with vitamin D levels, but not the GC heterozygous genotype, this may be attributed to possible conformational changes within the encoded protein impacted on by the minor alleles (when present in homozygous state). GC gene codes for a vitamin D-binding protein, and hence functional polymorphisms in this gene are expected to affect the overall function of this protein. Additionally, vitamin-D receptor’s polymorphisms may contribute to the vitamin levels, which would be a good future study too.

We used cut-off serum 25(OH)D levels of > 20 ng/ml as sufficient, of < 12 ng/ml as deficient, and of 12–20 ng/ml as insufficient to analyze the regression tree values. This cut-off chosen based on previously published data [22, 23]. Notably, most subjects in each group were vitamin D deficient, and the proportion of subjects with vitamin D sufficiency did not reach 30%, with exception of the subgroup D in which the majority of individuals (67%) is sufficient.

Among subjects who had lived in Kuwait for > 51 years, 22% had deficient vitamin D levels and 67% had sufficient levels, which contrasted with rs2298850-CC subjects who had lived in Kuwait for < 51 years, 70% of whom were deficient and 5% were sufficient. However, among rs2298850-GG individuals who had lived in Kuwait for < 51 years, 47% were deficient and 28% were sufficient, suggesting that other population-specific alleles or genes are involved in regulating vitamin D levels in Arab populations. These other genetic factors may explain the variance in vitamin D levels in rs2298850-GG individuals who have lived in Kuwait for < 51 years compared with those who have lived there for > 51 years. The identification of these genes or alleles would help to assess the risk of vitamin D deficiency in patients.

Notably, our results differ from those of a recent study [24] in which no evidence of any effect of the GC gene on vitamin D levels was found in subjects in Uzbekistan and Kazakhstan. Instead, that study showed an association with CYP2R1-rs10766197 and DHCR7/NADSYN1, which indicates population-specific genetic differences in the genes involved in the vitamin D pathway.

## Conclusions

Our analyses showed as the distribution of serum 25(OH)D levels in individuals with self-reported Arab ethnicity is skewed towards low values, highlighting a high prevalence of deficiency and insufficiency in vitamin D.

As demonstrated, common markers in GC and CYP2R1 gene affect the variation in vitamin D level in Arab individuals, nevertheless our conditional inference trees show that polymorphisms in GC can explain most of the variation in vitamin D level when compared with several other variants already reported associated to serum 25(OH)D levels, showing that this gene is the major player in Arab population, interacting with lifestyle features. One limitation is that only know variants were included in this study, future research effort should be focused in the sequencing of this gene in order to find population-specific rare deleterious variants with higher impact on serum 25(OH)D for a better assessment of vitamin D deficiency in Arab populations.

## Additional file

**Additional file 1: Table S1.** Gene and markers description used in this study.
