# Supplementary material for: Investigation of genetic variation and lifestyle determinants in vitamin D levels in Arab individuals
Source: J Transl Med. 2018 Jan 30;16:20. doi: 10.1186/s12967-018-1396-8 (PMC5791363; doi:10.1186/s12967-018-1396-8)
Supplement: Supplementary file 1 — Additional file 1: Table S1. Gene and markers description used in this study. [file 12967_2018_1396_MOESM1_ESM.docx]

**Table S1. Gene and markers description used in this study. Allele frequency from this study and 1000 G are also reported**

| SNP Id | Location | Consequence | CADD score | SYMBOL | Gene | Arabs | Africans | Americans | East Asians | Europeans | South Asians |
| --- | --- | --- | --- | --- | --- | --- | --- | --- | --- | --- | --- |
| rs17467825 | 4:71739800-71739800 | downstream_gene_variant | 3.739 | GC | ENSG00000145321 | 0.205 | 0.0545 | 0.2104 | 0.2609 | 0.2485 | 0.2975 |
| rs2282679 | 4:71742666-71742666 | intron_variant | 4.119 | GC | ENSG00000145321 | 0.202 | 0.0499 | 0.2089 | 0.2609 | 0.2465 | 0.2965 |
| rs3755967 | 4:71743681-71743681 | intron_variant | 5.763 | GC | ENSG00000145321 | 0.198 | 0.0545 | 0.2089 | 0.2609 | 0.2475 | 0.2975 |
| rs2298850 | 4:71748550-71748550 | intron_variant | 0.183 | GC | ENSG00000145321 | 0.189 | 0.0265 | 0.1974 | 0.2599 | 0.2356 | 0.2935 |
| rs7041 | 4:71752617-71752617 | missense_variant | 0.002 | GC | ENSG00000145321 | 0.585 | 0.0938 | 0.5375 | 0.2996 | 0.5835 | 0.5368 |
| rs1155563 | 4:71777771-71777771 | intron_variant | 0.556 | GC | ENSG00000145321 | 0.203 | 0.0575 | 0.1859 | 0.3363 | 0.2435 | 0.316 |
| rs206793 | 2:31450049-31450049 | intergenic_variant | 3.241 | - | - | 0.092 | 0.1256 | 0.0418 | 0.0565 | 0.0636 | 0.0112 |
| rs7116978 | 11:14860225-14860225 | intron_variant | 2.687 | PDE3B | ENSG00000152270 | 0.688 | 0.671 | 0.719 | 0.6796 | 0.6561 | 0.6585 |
| rs1993116 | 11:14888688-14888688 | intron_variant | 7.959 | CYP2R1 | ENSG00000186104 | 0.684 | 0.7814 | 0.7176 | 0.6796 | 0.6123 | 0.6472 |
| rs10500804 | 11:14888727-14888727 | intron_variant | 4.723 | CYP2R1 | ENSG00000186104 | 0.425 | 0.1029 | 0.5159 | 0.37 | 0.4483 | 0.4519 |
| rs12794714 | 11:14892029-14892029 | synonymous_variant | 12.26 | CYP2R1 | ENSG00000186104 | 0.427 | 0.1029 | 0.5144 | 0.3681 | 0.4473 | 0.4448 |
| rs10741657 | 11:14893332-14893332 | upstream_gene_variant | 0.206 | CYP2R1 | ENSG00000186104 | 0.687 | 0.7814 | 0.7161 | 0.6806 | 0.6193 | 0.6421 |
| rs7944926 | 11:71454579-71454579 | intron_variant | 3.695 | NADSYN1 | ENSG00000172890 | 0.424 | 0.1702 | 0.4496 | 0.381 | 0.7008 | 0.1513 |
| rs12785878 | 11:71456403-71456403 | intron_variant | 0.913 | NADSYN1 | ENSG00000172890 | 0.417 | 0.1702 | 0.4496 | 0.38 | 0.7008 | 0.1513 |
| rs4944957 | 11:71456989-71456989 | intron_variant | 0.631 | NADSYN1 | ENSG00000172890 | 0.447 | 0.4501 | 0.4813 | 0.381 | 0.7008 | 0.1513 |
| rs12800438 | 11:71459957-71459957 | intron_variant | 2.894 | NADSYN1 | ENSG00000172890 | 0.429 | 0.3321 | 0.4654 | 0.38 | 0.7008 | 0.1513 |
| rs3794060 | 11:71476633-71476633 | intron_variant | 0.558 | NADSYN1 | ENSG00000172890 | 0.421 | 0.1407 | 0.451 | 0.38 | 0.7008 | 0.1524 |
| rs3829251 | 11:71483513-71483513 | intron_variant | 3.844 | NADSYN1 | ENSG00000172890 | 0.237 | 0.2572 | 0.232 | 0.2976 | 0.1909 | 0.3538 |
| rs4945008 | 11:71510202-71510202 | upstream_gene_variant | 3.221 | RP11-684B2.3 | ENSG00000254924 | 0.413 | 0.1415 | 0.4496 | 0.38 | 0.6998 | 0.1503 |
